# Biomonitoring and assessment of toxic element contamination in floodplain sediments and soils using fluorescein diacetate (FDA) enzymatic activity measurements: evaluation of possibilities and limitations through the case study of the Drava River floodplain

**DOI:** 10.1007/s10661-022-10301-7

**Published:** 2022-08-03

**Authors:** Péter Szabó, Gyozo Jordan, Tamás Kocsis, Katalin Posta, Levente Kardos, Robert Šajn, Jasminka Alijagić

**Affiliations:** 1 Doctoral School of Environmental Sciences, ELTE Eötvös Loránd University, Budapest, Hungary; 2 Hungarian University of Agriculture and Life Sciences, Gödöllő, Hungary; 3 Geological Survey of Slovenia, Ljubljana, Slovenia

**Keywords:** Potentially toxic elements, Fluorescein diacetate activity, Heavy metals, Contamination, Biological activity

## Abstract

The EU Water Framework Directive requires the monitoring and evaluation of surface water sediment quality based on the assessment of risk posed by contamination on the biotic receptors. Floodplain sediments are important receptors of potentially toxic element (PTE) contamination from the upstream catchment areas, and floodplains host climate-sensitive riverine ecosystems and fertile agricultural areas at the same time. This study investigates the effect of PTE contamination on microbial communities in floodplain sediments and soils using the fast, inexpensive and reliable fluorescein diacetate (FDA) method in order to estimate its applicability for sediment quality monitoring and preliminary toxicity-based risk assessment. Sediment and soil samples were collected from the actively flooded alluvial plain and the river terrace areas along a 130-km stretch of the large Drava River floodplain known to be widely contaminated by historical mining, smelting and the associated industry in the upstream Alpine region. Results of detailed data analysis show that the total microbial activity represented by the measured FDA values is related to PTE (As, Cu, Zn, Cd, Pb) concentrations, but this relationship shows significant heterogeneity and depends on the spatial location and on the soil properties such as organic matter content, dissolved salt and nutrient content, and it is specific to the toxic elements. Results show that some microbe species appear to be able to adapt to the elevated PTE concentrations in toxic soil micro-environments, over time. Despite the observed heterogeneity of microbial activity, the results revealed a breakpoint in the FDA dataset around the FDA = 3 FC (fluorescein concentration) value suggesting that microbial activity is controlled by thresholds.

## Introduction

The EU Water Framework Directive (WFD) requires the monitoring and risk assessment of surface water sediment quality, in addition to the ongoing routine water quality monitoring (EC, 2010). According to the WFD, risk assessment and, therefore, the environmental limit values (environmental quality standards (EQSs)) must be based on the contaminants’ (hazardous substances; EC, 2008) toxic effect on the receptor pelagic or benthic biota (EC, 2018). This leads to the need of monitoring the biota associated with surface water sediments (EC, 2010). Although floodplain sediment is not in the scope of the WFD (surface water–suspended sediment and bottom sediment are considered only for monitoring), floodplain sediments are important indicators of contamination from the upstream catchment areas, on one hand, and host climate-sensitive riverine ecosystem and fertile agricultural receptors, on the other hand (EC, 2007; Salminen et al., 1998). Floodplain sediments are suspended sediments deposited during the short spells of flood events and are subject to soil formation as fluvisols immediately after flood retrieval (Hilscherova et al., 2007; Miller & Miller, 2007; Schwartz et al., 2006). Thus, the environmental quality assessment of floodplain sediments requires the monitoring of the receptor biota dwelling in the floodplain sediments and soils. Monitoring of biological activities in soils has been receiving increasing attention aiming at evaluating contaminant bioavailability and toxicity (Biró et al., 2014; Fekete et al., 2016; Toscano et al., 2014; Veres et al., 2015). The study of soil microbial activity offers an efficient method for monitoring and a number of methods for determining microbial abundance and population community in soils, such as integrated measurement of microbial counts, soil metabolic enzyme activities and soil respiration, in addition to the analysis of phospholipid fatty acids, which have been developed in order to assess soil degradation potential (Zhang et al., 2019; Kocsis et al., 2022). A sensitive and rapid method for measuring total microbial activity in soils is the fluorescein diacetate (FDA) test after Adam and Duncan (2001), modified after Schnürer and Rosswall (1982). The FDA hydrolysis assay measures the enzymatic activity of microbial populations and can provide an estimation for the overall microbial activity in a soil sample. The advantage of this method is its simplicity, sensitivity and rapidity (Dzionek et al., 2018; Schumacher et al., 2015). The FDA method has been optimised for soil science, and it is widely used in scientific and agricultural practices as well (Dick, 1997). Although there are many studies focusing on the optimisation of fluorescein diacetate assays on different types of soils (Adam & Duncan, 2001; Green et al., 2006; Schumacher et al., 2015), its utilisation to assess the effects of toxic element pollution in floodplains and riverine systems is still poorly studied (Jaiswal & Pandey, 2019; Kumar et al., 2019; Liu et al., 2018). Jiang et al. (2016) measured the FDA hydrolysis intensity using sediments treated with dissolved inorganic nitrogen, phenanthrene and cadmium. They found that fluorescein was generated from the sediments with the addition of nitrogen and phenanthrene. On the contrary, the addition of Cd depressed the microbial activity. Moreover, it has been found that the speciation of chemical elements and the grain size determine PTE bioavailability and thus their toxicity in soils (Alloway, 2012; Byrne et al., 2012; Contin et al., 2007; Du Laing et al., 2009; Enya et al., 2020; Hindersmann & Mansfeldt, 2014; Hodson, 2012; Kabata-Pendias & Pendias, 2001; Tolar et al., 2020; Young, 2013). Kumar et al. (2019) have shown that FDA in the bed sediment can be used as an ecosystem ‘response’ to carbon, nutrients and metal pollution in human-impacted rivers. The FDA activity showed dependence on substrates (carbon and nutrients) when the heavy metal concentrations are below the toxic threshold. Jaiswal and Pandey (2019) confirmed a similar conclusion. Their results emphasise the importance of FDA as a response determinant of ecosystem functional shifts in large rivers. They studied simultaneously the FDA activity, the total organic carbon and the microbial biomass-derived carbon (*C*_mic_) content with the total metal concentration in an empirical relationship to provide a quantitative diagnostic indicator for the eutrophy/metal-pollution responses in human-impacted rivers.

Previous studies (Halamić et al., 2003; Peh et al., 2008; Šajn et al., 2011) have shown that soils and sediments are contaminated along the nearly 300-km-long trans-boundary (Austria, Slovenia, Croatia, Hungary) floodplain of the Drava River which, in turn, embraces important agricultural lands and rich riverine habitats (Lieb & Sulzer, 2019). The Drava is one of the longest rivers in Europe (725 km) draining a large trans-boundary catchment (42,240 km^2^). The vicinity of the river is inhabited since ancient times. Important historical mines, smelters and industry centres are located in the catchment area, polluting the river floodplain sediments and soils with potentially toxic elements (PTEs) (ATSDR, 2019) such as As, Cu, Zn, Cd and Pb for a long time (Halamić et al., 2003; Peh et al., 2008; Šajn et al., 2011).

The general objective of this study is to investigate the effect of PTE contamination on microbial communities in floodplain sediments and soils using the fast and reliable FDA method in order to estimate its applicability for sediment quality monitoring and toxicity-based risk assessment. The specific objective is to get an insight into the soil microbial conditions in the PTE-contaminated Drava River floodplain and therefore assist land use and soil resources planning. The hypothesis tested is that FDA can reflect PTE contamination levels in two different depths (topsoil and subsoil) of the alluvial plain and river terrace areas, as well as the land cover types influenced by the Drava River.

## Materials and methods

### Study area

**Fig. 1:**
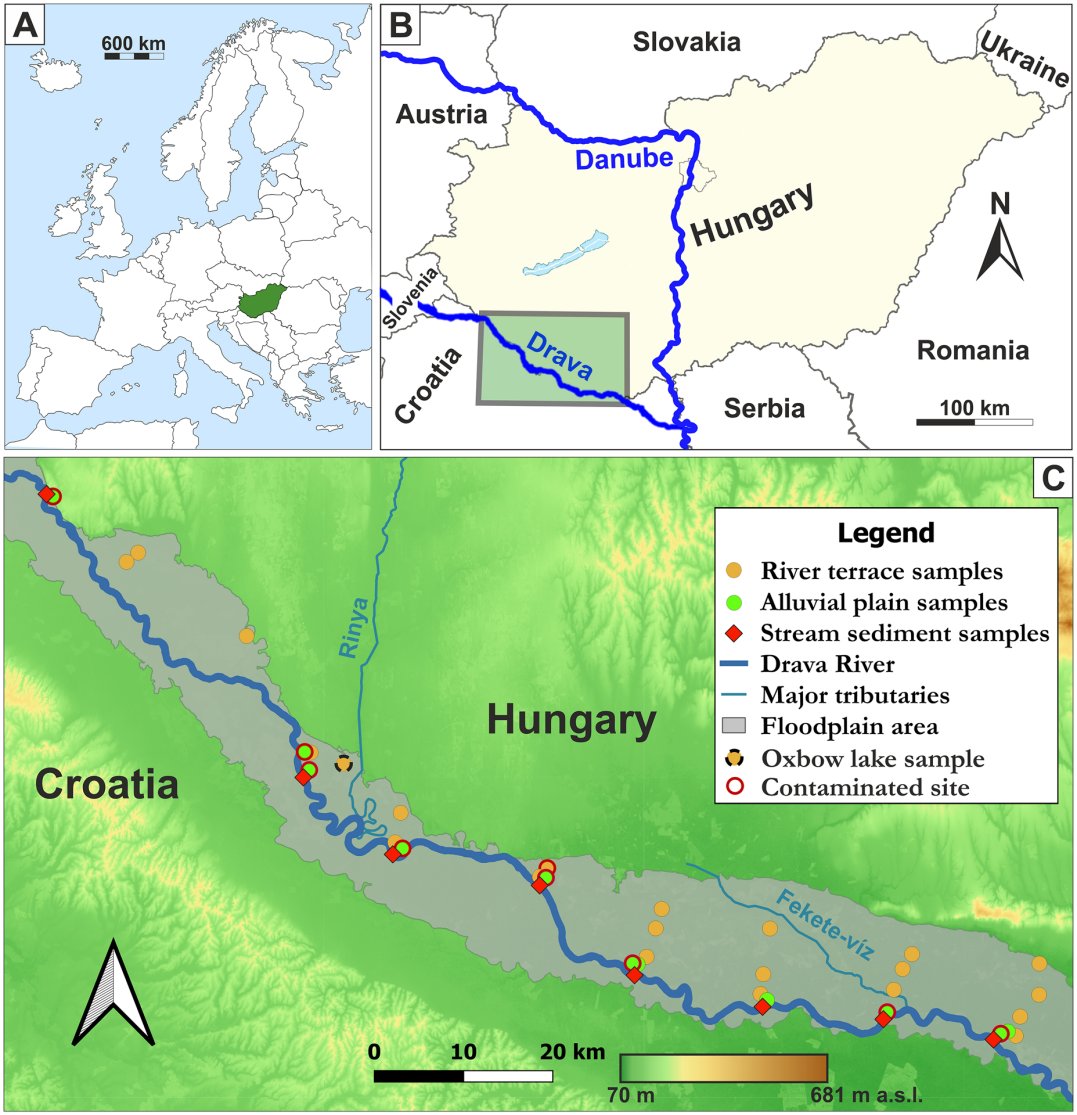
**A** Regional orientation map for the study area. **B** The trans-boundary location of the Drava River draining into the Danube River. The studied floodplain area is shown by the grey box. **C** Location of sampling sites along the studied floodplain. Location of map is shown in **B**

Drava is the third largest river in the Carpathian Basin, Europe (749 km), with a unique and diverse riverine ecosystem. Its source area is in the Alps in San Candido, South Tirol, in northern Italy, and it runs across Austria, Slovenia, Hungary and Croatia, discharging into River Danube eventually (Fig. 1). The 140-km section investigated in this study runs along the Hungarian-Croatian border. Hydromorphologically, under the prevailing continental climate conditions, the river is surrounded by regularly (1–2 times a year) flooded alluvial plains characterised by dense riverine forest vegetation, and slightly elevated river terraces (inactive old alluvial plains) located further away covered mostly with agricultural lands (Lieb & Sulzer, 2019; Fig. 1). The construction of continuous 140-km-long embankments along the Drava River began in the early 1740s; however, until the river regulation works took place and the embankments were finished in the 1880s, the majority of the Drava River floodplain area (ca. 115,000 ha) was periodically flooded (Buchberger, 1975; Remenyik, 2004, 2005). River regulation activities also included 64 cutoffs, which resulted in several oxbow lakes in the studied floodplain area (Brewer & Taylor, 1997; Ihrig, 1973; Schwarz, 2019). Upstream of the studied floodplain section, 22 hydropower plants were constructed between 1918 and 1989 (upstream from the confluence of the rivers Drava and Mur) in Austria, Slovenia and Croatia (Bonacci & Oskoruš, 2008; Burián & Domány, 2019).

Settlements and some industries are found on the floodplain along the river, influencing the chemical composition of the sediments and soils, but historical mining and smelting in the upstream Alpine region are the most important contamination sources (Halamić et al., 2003; Peh et al., 2008; Šajn et al., 2011). The mining activities originate back to ancient times, culminating in the last century and resulting in elevated As, Cu, Zn, Cd and Pb concentrations in the area. The position of the riverbed has been constantly changing over time that is why the historical contamination can be found in the sediments and soils over a wide area (Šajn et al., 2011). The prevailing land cover in the Drava catchment draining to the studied river section (see Fig. 1) is the agricultural areas (60.4%), which consist of arable lands (51%), heterogeneous agricultural areas (5%), pastures (4.1%) and permanent crops (0.3%). The second largest land cover type is the forests and seminatural areas (36.4%), followed by artificial surfaces (2.9%) and inland wetlands (0.3%) (CLC, 2018).

### Sampling

Sampling design and sample collection followed the method of Šajn et al. (2011), which was based on the Field Manual (‘Green Book’) of the FOREGS European Geochemical Mapping Project (Salminen et al., 1998, 2005), in order to be consistent with the previous research results from the upstream parts of the Drava River floodplain. Accordingly, soil and sediment samples were collected from the alluvial plains and the river terraces along transects across the Drava River located at 10-km intervals, in April 2017 (Fig. 1). At each sampling point, topsoil (0–10 cm) and subsoil (20–30 cm) samples were collected, composed of 5 sub-samples located at the corners and middle of a minimum 10 × 10 m plot, 4 kg of weight each. Riverbed sediment samples were collected in the temporarily dry riverbed at base flow conditions (low water level in April) and were composed of 5 sub-samples, giving out altogether 4 kg per sample. The collected 8 river bed sediment samples were not analysed for microbial activity; therefore, these samples are not considered in this study further. Floodplain soil sampling targeted two different hydromorphological regimes with characteristic soil types: the actively flooded alluvial plain with fluvisol under the dense riparian forests and the historically flooded river terrace having a combination of fluvisol and cambisol, which are now widely used for agriculture activities (Lieb & Sulzer, 2019; Šajn et al., 2011). In this way, altogether 22 soil samples were taken on the actively flooded areas at 11 points and 44 on the river terraces at 22 points (Fig. 1).

### Laboratory analyses

#### Element concentration measurements

The collected soil and sediment samples were dried at room temperature until constant weight and manually disaggregated as needed. After thorough homogenisation and sieving to the < 2-mm fraction, samples were pulverised to 0.63 μm (Salminen et al., 1998). The chemical analysis was carried out by the ACME Ltd. laboratories in Vancouver, Canada, using inductively coupled plasma mass spectrometry after four acid digestion (HClO_4_, HNO_3_, HCl and HF at 200 °C) (Šajn et al., 2011). The following chemical elements are discussed in this manuscript: As, Cu, Zn, Cd and Pb as PTEs and P as an essential nutrition element for microbial activity.

#### Soil parameter measurements (organic matter content, electrical conductivity, pH, carbonate content, grain size)

Small portions (~ 40 g) of the collected soil and sediment samples processed in the above-described way (drying, homogenisation, sieving) were used for organic matter content, electrical conductivity (EC), pH and carbonate content measurements. Organic matter content was approximated by loss on ignition (LOI) method on approximately 2-g sample test portion at ~ 600 °C temperature until reaching constant weight (MSZ, 2013). Carbonate content measurement used the Scheibler gas volumetric method on 2-g sample (MSZ, 1978) and pH measurement used the deionised water leaching method on 5-g sample. After 24-h leaching time, pH was measured by an ADWA AD 14 field pH metre (MSZ, 1981). Electrical conductivity (EC), representing soluble soil salt content, was measured in 5-g soil sample suspension using deionised water by an ADWA AD 32 field EC/TDS metre (MSZ, 1981). For grain size analysis, first 80–150-g dry sample was wet sieved through a standard mesh size series. The different fractions were collected separately, dried in 105 °C until constant weight and measured. The < 0.063-mm fraction was collected in a 20-L vessel, left for settling, and it was dried as well after removing the excess water. After drying, a small amount was taken out and analysed with laser diffractometry (MSZ, 1979).

#### Soil microbiological activity

Unless otherwise indicated, the method of Schnürer and Rosswall (1982) was followed for the FDA activity estimation, with some modifications by Adam and Duncan (2001). The FDA is a cell-permeant esterase substrate that can serve as a viability probe that measures both enzymatic activity, which is required to activate its fluorescence, and cell-membrane integrity, which is required for intracellular retention of their fluorescent product. The method has been used for monitoring the soil microbiological activity by measuring the total catabolic enzyme activity of soil microorganisms. Two grammes of Drava floodplain soil sample (particle size < 300 microns) was measured in three repetitions. For each sample, 2 g of sample was measured into each of the three 50-ml falcon tubes. In every case, one out of the three 2 g of sample was added into the tube, which served as the ‘No-FDA’ control (sample without FDA indicator). In another preparation, 20 ml of 60 mM phosphate buffer was added to one of the tubes. This was another control containing FDA without soil sample (blind). One hundred microliters of 2000 µg ml^−1^ stock FDA solution was added to each of the tubes except for the ‘No-FDA’ control sample. The resulted concentration of FDA was 10 µg ml^−1^. The samples were shaken at 200 rpm at 30 °C. After 60 min, the reaction was stopped with 20 ml of 2:1 ratio chloroform:methanol mix in each tube.

In the FDA assay, the samples were mixed with FDA buffer, incubated and shaken for 1 h. The intensity of the resulting yellow-green colour was indicative of the amount of enzymatic cleavage of the FDA molecule and the overall enzymatic activity in the sample. Quantification of enzyme activity was performed by assessing the intensity of colour formation measuring the absorbance at 490 nm using Biochrom Libra S22 spectrophotometer. Values are given as microlitres of fluorescein in 1 g of soil after 1 h of incubation period (μlgFl/h) according to Adam and Duncan (2001). The least significant differences (LSD5%) were estimated by conventional variance analysis. FDA results are given in units of fluorescein concentration (FC).

### Statistical data analysis

Central tendency and variability measures of the chemical element concentrations, soil parameters and FDA values used in this study were the minimum, lower quartile, median, upper quartile, maximum, median absolute deviation (MAD), average (arithmetic mean) and standard deviation (Kurzl, 1988). For outlier identification, Tukey’s (1977) inner fence criteria was used. Accordingly, outliers and extreme values are located at a distance of 1.5 times and 3 times the interquartile range from the lower or upper quartile, respectively (Davis, 2002; Reimann et al., 2008). Sub-population identification followed the ‘natural break’ histogram slicing method (Abdaal et al., 2013). A natural break is defined in the cumulative distribution function at an inflection point, identified visually on the cumulative distribution function curve. This point corresponds to a local minimum in the frequency histogram.

Bivariate data analysis included the calculation of Pearson’s linear correlation coefficient and fitting the least squares regression line to the data points. All discussed results are significant at the 95% confidence level.

Principal component analysis (PCA) was performed on the soil parameters, as a dimensionality reduction procedure, to find the components (linear combination of variables) influencing the variability of the dataset. The original dataset was projected into principal components, using an orthogonal linear transformation, preserving the variation of the data as much as possible (Davis, 2002; Reimann et al., 2008). The original parameters were normalised between 1 and 100 to facilitate the comparison among the parameters with different measurement units and orders of magnitude. The principal components with an eigenvalue less than 1 were not taken into consideration. The principal component analysis creates artificial parameters by combining the original variables; thus, the significant geochemical processes that have an influence on the samples can be revealed.

Statistical data analysis was performed using STATGRAPHICS Centurion XVIII software.

## Results

### Floodplain soil chemistry characterisation

**Fig. 2:**
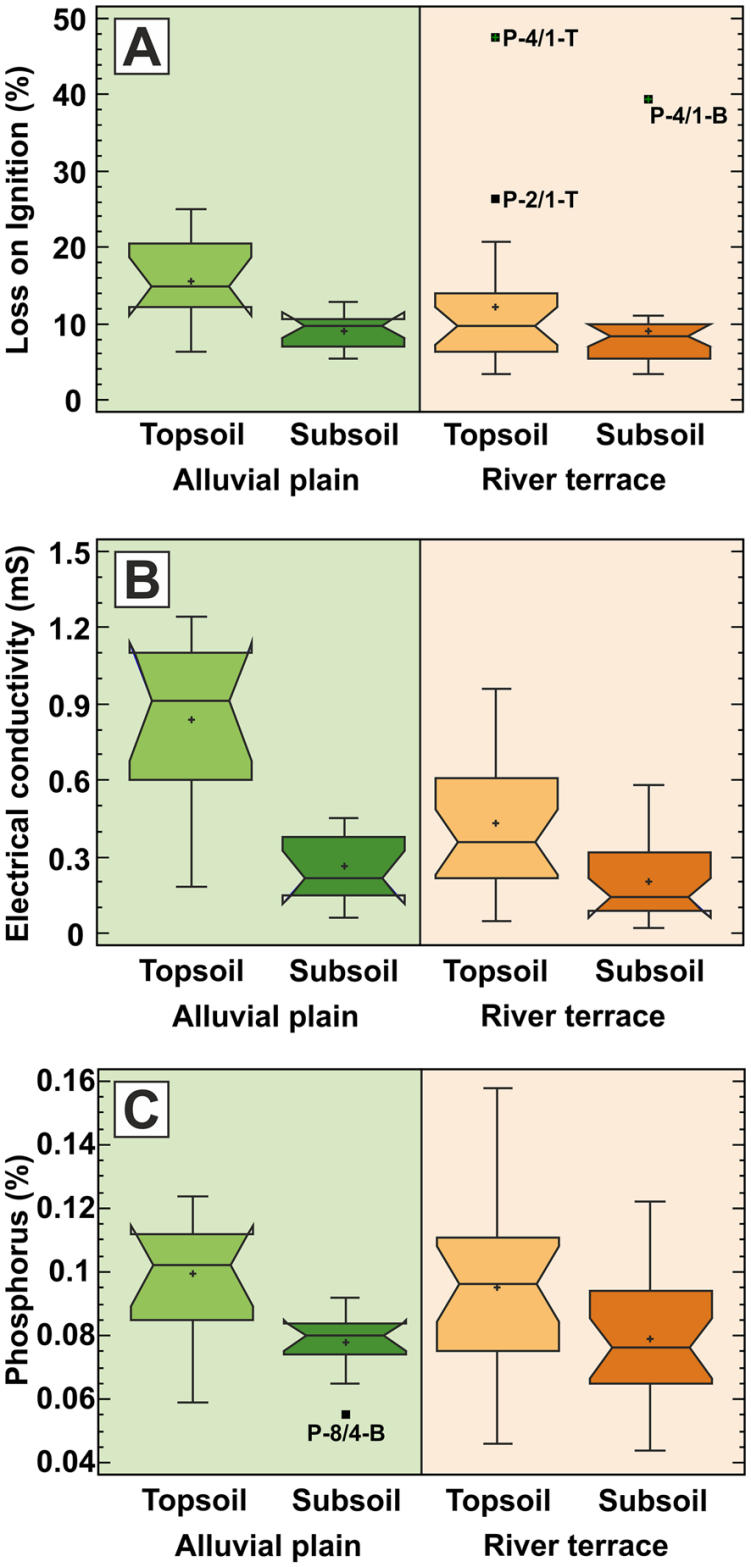
Box plot charts for **A** organic matter content (loss on ignition (LOI)), **B** electrical conductivity (EC) and **C** phosphorus (P), showing the differences between the alluvial plain (green shading) and river terrace (brown shading) areas, together with the differences between the topsoil and subsoil sampling horizons. The outlying and extreme values are marked with solid black rectangles and with their sample identification codes

Box plots showing graphically the basic statistical parameters in Table 1 reveal that LOI, EC and P tend to have higher median values and variability in topsoils than in subsoils both in the active alluvial plain and in the old river terraces (Fig. 2). In fact, their values are very similar among the alluvial plain and river terrace topsoil samples, and among the alluvial plain and river terrace subsoil samples, showing that past and recent flooding events have little effect on their distribution. Thus, the geochemical distribution of LOI, EC and P is related to the soil horizons rather than to the geomorphological setting (active alluvial plain vs agricultural river terrace). Assuming that subsoil samples represent the geochemical background, it can be seen that these parameters have uniformly low median and variability values in the whole floodplain area irrespective of the geomorphological setting (alluvial plain vs river terrace) (Fig. 2). Altogether, soil parameters LOI, EC and P have significantly higher values in topsoils than in subsoils, as also confirmed by the Mann–Whitney test at the 95% confidence level. It is noted that the observed pattern of LOI, EC and phosphorus distribution is very similar to that of the FDA microbial activity (compare Figs. 2 and 6).

**Table 1:** Basic statistical parameters of the studied soil parameters (LOI, EC, pH, CaCO_3_, P), also showing FDA (*Min* minimum, *LQ* lower quartile, *UQ* upper quartile, *Max* maximum, *MAD* median absolute deviation, *Avg* average, *SD* standard deviation, *MAD/median* relative variability, *TS* topsoil, *BS* subsoil, *AP* alluvial plain, *RT* river terrace)

	**Locality**	**LOI**	**EC**	**P**	**pH(water)**	**CaCO**_**3**_	**FDA**
		*%*	*mS*	*%*	*–*	*%*	*FC*
**Min**	**Overall**	3.35	0.02	0.044	5.41	0.00	0.65
	**TS**	3.46	0.05	0.046	5.41	0.00	0.65
	**BS**	3.35	0.02	0.044	5.68	0.00	0.67
	**AP**	5.39	0.06	0.055	6.52	0.31	0.67
	**RT**	3.35	0.02	0.044	5.41	0.00	0.65
**LQ**	**Overall**	6.44	0.14	0.073	6.88	0.21	1.58
	**TS**	6.79	0.32	0.079	6.88	0.21	2.21
	**BS**	6.40	0.11	0.066	6.90	0.31	1.18
	**AP**	7.89	0.18	0.079	6.91	0.73	1.76
	**RT**	5.75	0.12	0.071	6.75	0.10	1.57
**Median**	**Overall**	9.74	0.33	0.086	7.27	0.52	2.50
	**TS**	11.82	0.51	0.098	7.21	0.42	6.96
	**BS**	8.55	0.18	0.079	7.36	1.05	1.77
	**AP**	11.17	0.40	0.085	7.30	1.36	5.33
	**RT**	8.73	0.28	0.088	7.22	0.31	2.22
**UQ**	**Overall**	12.82	0.52	0.102	7.42	1.78	7.23
	**TS**	16.30	0.91	0.111	7.32	1.05	9.94
	**BS**	10.18	0.36	0.092	7.58	2.31	2.55
	**AP**	14.85	0.91	0.102	7.40	2.20	9.68
	**RT**	10.88	0.45	0.103	7.49	1.00	5.36
**Max**	**Overall**	47.40	1.24	0.158	7.77	9.45	14.65
	**TS**	47.40	1.24	0.158	7.61	2.83	14.65
	**BS**	39.30	0.58	0.122	7.77	9.45	8.04
	**AP**	25.01	1.24	0.124	7.71	3.67	14.65
	**RT**	47.40	0.96	0.158	7.77	9.45	12.58
**MAD**	**Overall**	3.21	0.19	0.013	0.25	0.42	1.46
	**TS**	5.03	0.29	0.014	0.21	0.32	3.69
	**BS**	1.75	0.11	0.013	0.24	0.95	0.67
	**AP**	3.29	0.25	0.011	0.12	0.69	4.10
	**RT**	2.35	0.17	0.016	0.30	0.21	1.11
**Avg**	**Overall**	11.12	0.40	0.088	7.08	1.19	4.50
	**TS**	13.28	0.57	0.097	7.00	0.81	6.76
	**BS**	8.96	0.22	0.078	7.16	1.57	2.24
	**AP**	12.33	0.55	0.089	7.21	1.50	5.78
	**RT**	10.52	0.32	0.087	7.02	1.03	3.86
**SD**	**Overall**	7.70	0.32	0.225	0.53	1.63	3.86
	**TS**	8.67	0.35	0.024	0.53	0.86	4.14
	**BS**	5.98	0.15	0.016	0.52	2.08	1.61
	**AP**	5.21	0.39	0.019	0.32	0.91	4.56
	**RT**	8.68	0.25	0.024	0.60	1.88	3.33
**MAD/median**	**Overall**	0.33	0.57	0.15	0.03	0.81	0.58
	**TS**	0.43	0.57	0.14	0.03	0.76	0.53
	**BS**	0.21	0.61	0.16	0.03	0.90	0.38
	**AP**	0.29	0.63	0.13	0.02	0.50	0.77
	**RT**	0.27	0.59	0.18	0.04	0.68	0.50

**Fig. 3:**
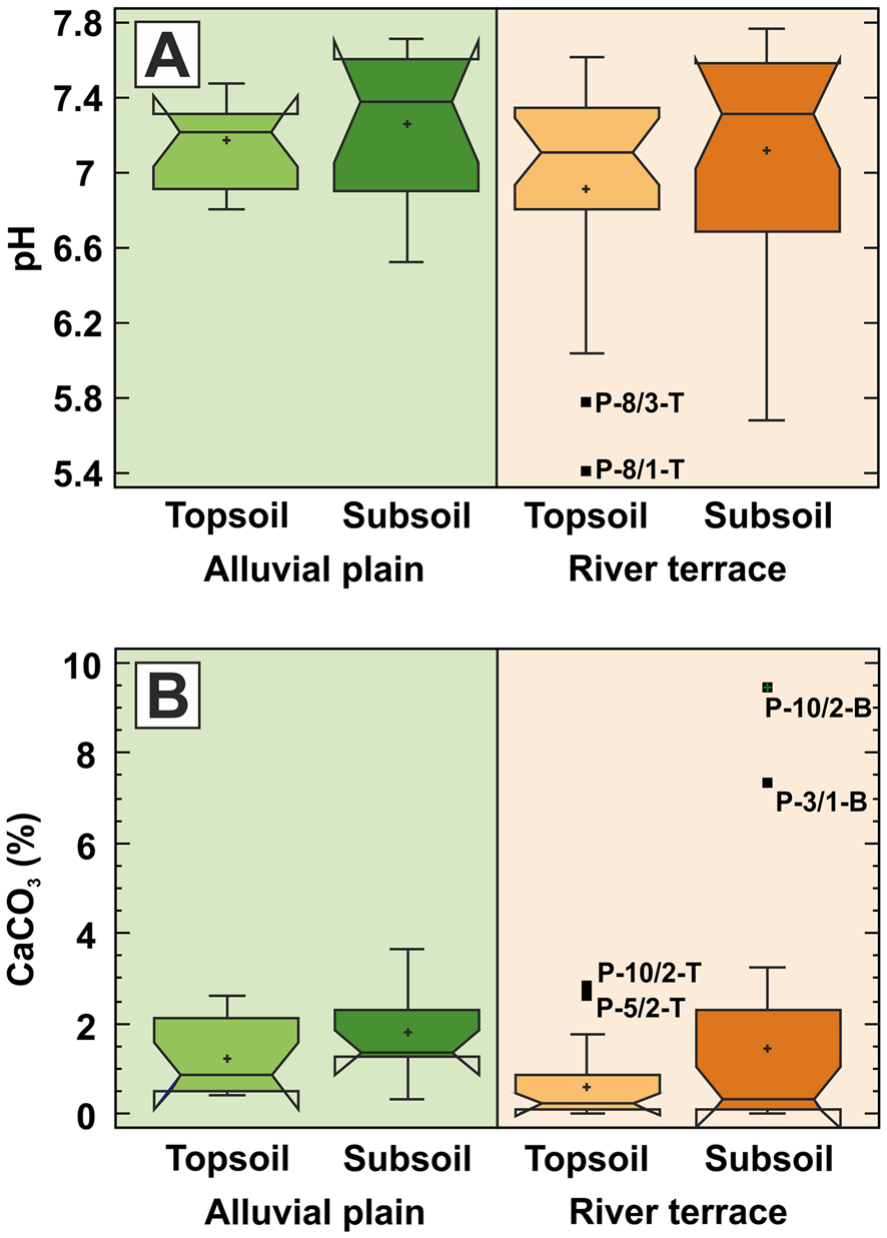
Box plot charts for **A** soil acidity (pH) and **B** total carbonate content (CaCO_3_), showing the differences between the alluvial plain (green shading) and river terrace (brown shading) areas, together with the differences between the topsoil and subsoil sampling horizons. The outlying and extreme values are marked with solid black rectangles and with their sample identification codes

Unlike LOI, EC and P, the measured values of soil pH and carbonate content are uniformly distributed throughout the studied floodplain soils and sediments irrespective of the soil horizon and geomorphological setting. According to Mann–Whitney test, pH has a uniform median value of around 7.3 in the whole study area, irrespective of the geomorphological environment or soil depth. pH seems to be more variable with slightly higher value in the studied subsoils (Fig. 3). Carbonate content has similar uniform distribution in the analysed soil and sediment samples, being more variable with slightly higher concentration values in the studied subsoils, too (Fig. 3). The somewhat depleted carbonate content in river terrace topsoils is due to agricultural soil cultivation in this area.

Regarding the potentially toxic elements, it can be seen in Table 2 that, apart from copper, all of the studied potentially toxic elements exceed the environmental threshold (environmental quality standard (EQS)) in one or more samples showing that the studied Drava floodplain is widely contaminated.

**Table 2:** Basic statistical parameters of the total chemical element concentration after four-acid digestion (*Min* minimum, *LQ* lower quartile, *UQ* upper quartile, *Max* maximum, *MAD* median absolute deviation, *Avg* average, *SD* standard deviation, *MAD/median* relative variability, *TS* topsoil, *BS* subsoil, *AP* alluvial plain, *RT* river terrace). Bold: values exceeding the EQS threshold (Decree 6/2009, 2009). Italics: values exceeding the FOREGS geochemical baseline concentrations (Salminen et al., 1998)

	**Locality**	**As**	**Cu**	**Zn**	**Cd**	**Pb**
		*mg/kg*				
	**Overall**	*6*	*12.6*	*48*	0.1	*17.7*
**Min**	**TS**	*7*	*13.1*	*48*	0.1	*18.3*
	**BS**	*6*	*12.6*	*52*	0.1	*17.7*
	**AP**	*6*	*12.6*	*156*	*0.6*	*61.6*
	**RT**	*6*	*13.1*	*48*	0.1	*17.7*
	**Overall**	*12*	*22.6*	*77*	*0.2*	*23.3*
**LQ**	**TS**	*12*	*22.8*	*82*	*0.2*	*23.4*
	**BS**	*13*	*22.6*	*77*	*0.2*	*23.3*
	**AP**	*13*	*22.8*	*197*	*0.9*	*73.5*
	**RT**	*12*	*22.2*	*72*	*0.2*	*21.55*
	**Overall**	***15***	*28.8*	*107.5*	*0.3*	*33.35*
**Median**	**TS**	***15***	*30.8*	*106*	*0.3*	*33.4*
	**BS**	***15***	*28.2*	*109*	*0.3*	*31.8*
	**AP**	***15.5***	*30.4*	***347.5***	***1.3***	***130.75***
	**RT**	***15***	*28.1*	*84.5*	*0.25*	*27.05*
	**Overall**	***19***	*37.2*	*197*	*0.9*	*83.1*
**UQ**	**TS**	***17***	*35.9*	*196*	*0.9*	*84.5*
	**BS**	***19***	*37.8*	*197*	*0.7*	*81.2*
	**AP**	***17***	*34.4*	***489***	***1.9***	***192.3***
	**RT**	***19.5***	*38.4*	*107.5*	*0.3*	*33.35*
	**Overall**	***36***	*48.5*	***854***	***3***	*232.3*
**Max**	**TS**	***25***	*48.5*	***854***	***3***	***232.3***
	**BS**	***36***	*47.2*	***784***	***2***	***194.5***
	**AP**	***20***	*40.3*	***854***	***3***	***232.3***
	**RT**	***36***	*48.5*	*196*	*0.8*	*89.4*
	**Overall**	3	7.05	45.5	0.1	13.35
**MAD**	**TS**	3	6.4	49	0.1	12.5
	**BS**	4	7.2	39	0.2	13.6
	**AP**	2	4.75	146	0.5	58.85
	**RT**	4	8.35	19.5	0.05	5.8
	**Overall**	***15.47***	*29.21*	*186.94*	*0.64*	*63.21*
**Avg**	**TS**	*14.91*	*29.50*	*199.85*	*0.77*	*67.24*
	**BS**	***16.03***	*28.93*	*174.03*	*0.54*	*59.18*
	**AP**	***15.05***	*28.86*	***380.18***	***1.44***	***128.53***
	**RT**	***15.68***	*29.39*	*90.32*	*0.26*	*30.55*
	**Overall**	5.21	9.86	181.33	0.71	57.96
**SD**	**TS**	4.22	9.34	201.85	0.83	64.37
	**BS**	6.05	10.50	160.31	0.52	51.43
	**AP**	3.55	8.06	202.51	0.71	56.83
	**RT**	5.89	10.73	31.88	0.13	15.01
	**Overall**	0.20	0.24	0.42	0.33	0.40
**MAD/median**	**TS**	0.20	0.21	0.46	0.33	0.37
	**BS**	0.27	0.26	0.36	0.67	0.43
	**AP**	0.13	0.16	0.42	0.38	0.45
	**RT**	0.27	0.30	0.23	0.20	0.21
**FOREGS**		*6*	*12*	*48*	*0.145*	*15*
**Decree 6/2009 **(2009)		**15**	**75**	**200**	**1**	**100**

**Fig. 4:**
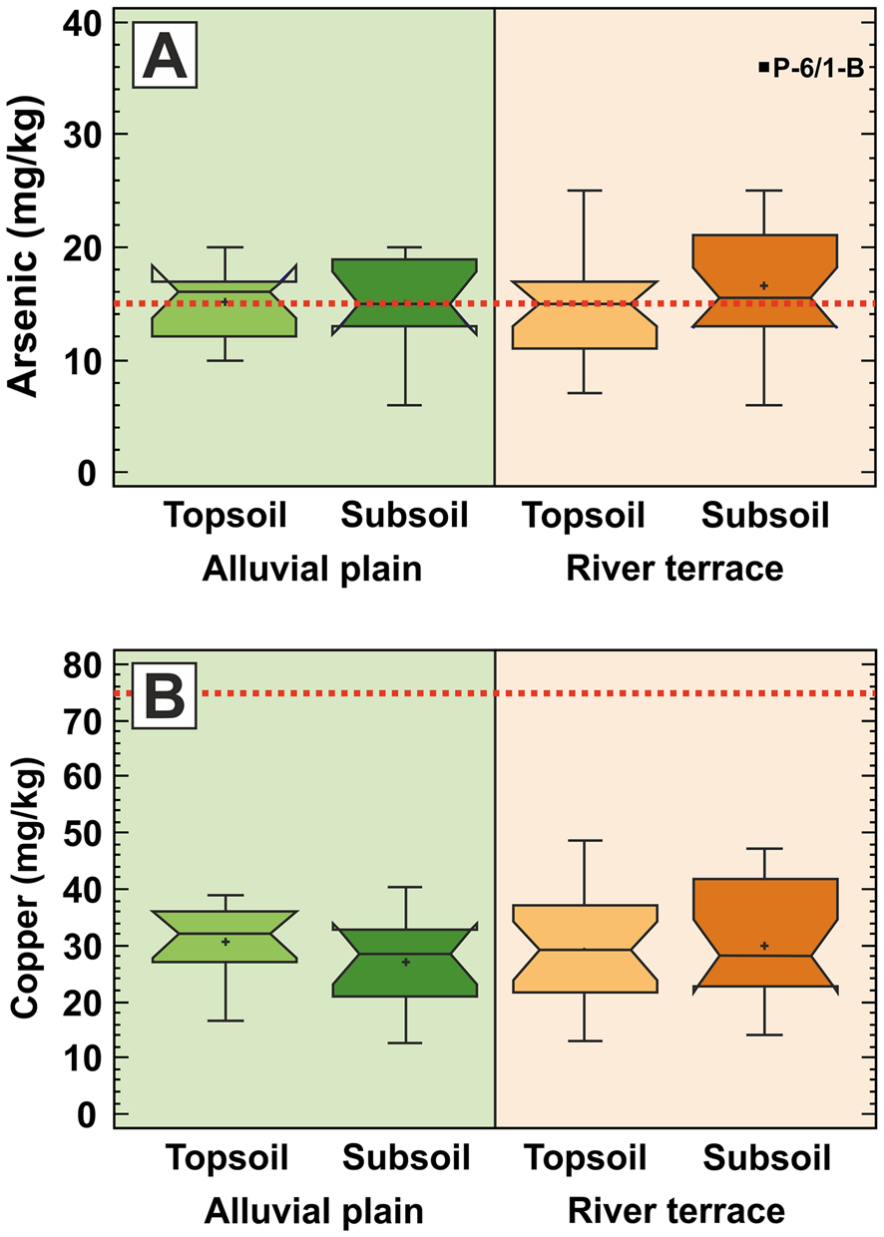
Box plot charts for **A** arsenic and **B** copper, showing the differences between the alluvial plain (green shading) and river terrace (brown shading) areas, together with the differences between the topsoil and subsoil sampling horizons. The outlying and extreme values are marked with solid black rectangles and with their sample identification codes. Dotted red lines show the EQS values for As (15 mg/kg) and Cu (75 mg/kg), respectively

According to the box plots in Fig. 4, arsenic and copper are almost uniformly distributed over the whole floodplain as shown by the similar median values (median As concentration: ~ 15 mg/kg; median Cu concentration: ~ 30 mg/kg), as confirmed by the Mann–Whitney test, and by the similar variability values (MAD As concentration: ~ 3 mg/kg; MAD Cu concentration: ~ 7.05 mg/kg), irrespective to the soil horizon and geomorphological setting. While Cu remains well below its EQS threshold value (75 mg/kg) everywhere in the studied floodplain, As is mostly above the EQS value (15 mg/kg) all over the investigated Drava River floodplain area (Fig. 4; Table 2). These results suggest that As and Cu distribution is defined by a process, acting in the past (river terrace) and recently (alluvial plain) in the same way and affecting topsoils and subsoils equally, irrespective of agricultural activities (terrace topsoil), concurrent flood events (alluvial plain topsoil) and being insensitive to oxidising (terrace) and reducing (alluvial plain) conditions. All these point to that the observed As and Cu concentrations represent the natural geochemical background of the river catchment.

**Fig. 5:**
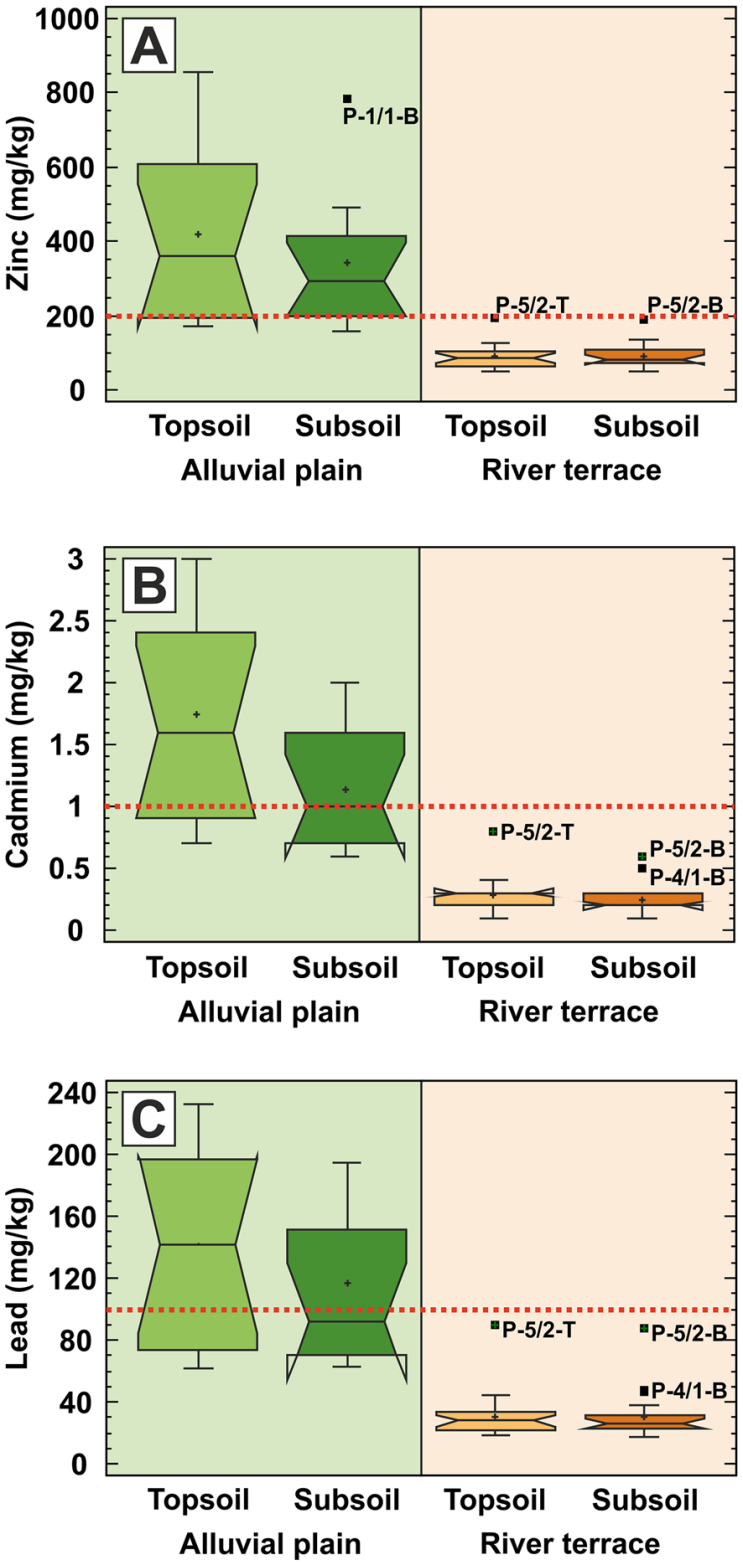
Box plot charts for **A** zinc, **B** cadmium and **C** lead, showing the differences between the alluvial plain (green shading) and river terrace (brown shading) areas, together with the differences between the topsoil and subsoil sampling horizons. The outlying and extreme values are marked with solid black rectangles and with their sample identification codes. Dotted red lines show the EQS values for Zn (200 mg/kg), Cd (1 mg/kg) and Pb (100 mg/kg)

The multiple box plots in Fig. 5 show very similar distribution pattern of Zn, Cd and Pb, which are markedly different from the previously seen patterns of As and Cu. The immediate and most striking feature is that the concentrations of these metals in the actively flooded alluvial plain are much higher both in the topsoil and in the subsoil horizons than the corresponding concentrations in the river terrace area. In fact, Zn, Cd and Pb concentrations are above the EQS limit values (see dashed lines in Fig. 5) in almost all samples in the alluvial plain, while they all remain well below the environmental threshold values in the terrace samples. Not only the overall (median) concentration values but the variability of the measured concentrations is much higher in the active alluvial plain, too (Fig. 5): median concentrations tend to be 4.1, 5.2 and 4.8 times higher, and total variability measured by the concentration range tend to be 4.7, 3.4 and 2.8 times higher in the active alluvial plain than in the terrace area, respectively. This shows that the hydrological regime has a significant impact on the Zn, Cd and Pb contamination distribution and the driving contamination process affects only the recently flooded areas irrespective to the soil horizon. The few outliers are associated with one sampling location in an agricultural area and another one located in an oxbow in the river terrace area (see Fig. 1).

In summary, the studied potentially toxic elements seem to have 2 distinct groups. As and Cu are uniformly distributed in the whole floodplain irrespective of the geomorphological setting and the soil horizon, while Zn, Cd and Pb have an identical and characteristic pattern of uniformly high and low concentrations in the alluvial plain and the river terrace, respectively.

**Fig. 6:**
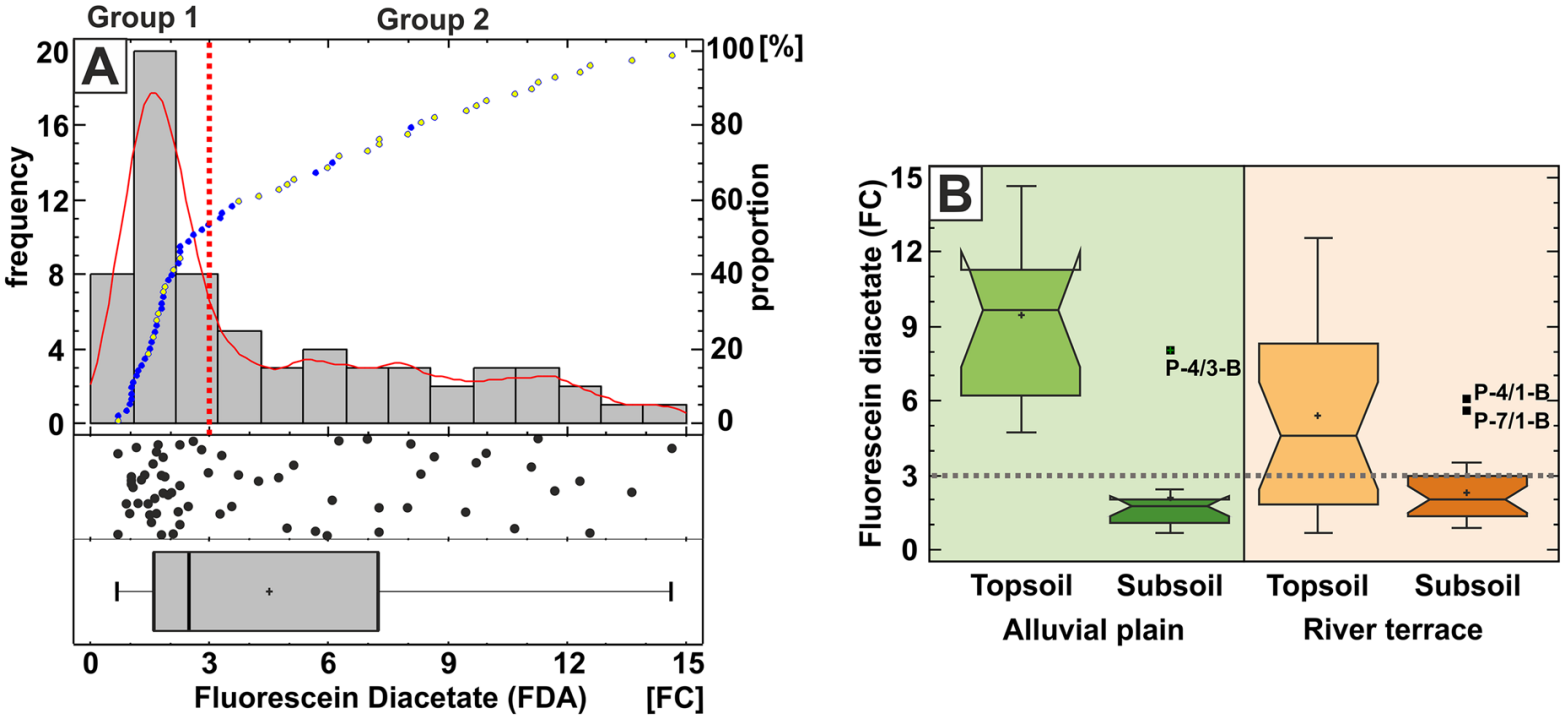
**A** One-variable analysis of the fluorescein diacetate (FDA) activity values. Note the two distinct groups separating topsoil samples (yellow dots) and subsoil samples (blue dots) above and below the FDA = 3 FC value (red dotted line). **B** Box plot chart of the fluorescein diacetate (FDA) values showing the differences between the alluvial plain (green shading) and river terrace (brown shading) areas, together with the differences between the topsoil and subsoil sampling horizons. The outlying and extreme values are marked with solid black rectangles and with their sample identification codes. Grey dotted line shows the FDA = 3 FC value. See text for details

The bulk microbial activity in the studied floodplain sediments as measured by FDA activity, ranges from 0.649 FC to 14.89 FC. The histogram of FDA clearly reveals a break in the dataset around the fluorescein diacetate activity value 3 FC, separating the dataset into two statistically significant different groups, as also confirmed by Mann–Whitney test (Fig. 6A). The lower group consists of subsoil samples and the upper group is made up of topsoil samples, predominantly. The studied topsoils have 4 times higher median FDA values than the subsoils and the bulk microbial activity within topsoil samples is higher in the active alluvial plain than in the river terrace, in general. In addition, the microbial activity in the topsoils is 1.4 times more variable than that in the subsoils, as measured by MAD/median relative variability index. This indicates the higher heterogeneity of microbial activity in near-surface soils, especially in the actively flooded alluvial plain area. FDA bulk microbial activity seems to be related to the soil horizon, rather than to the geomorphological setting (active alluvial plain vs river terrace). It is noted that the distribution pattern of the FDA microbial activity is very similar to that of the LOI, EC and phosphorus (compare Figs. 2 to 6). However, a few high outliers are present in subsoils, as well, drawing attention to heterogeneities in the deeper soil horizon.

### Relationship between FDA microbial activity and PTE distribution

**Fig. 7:**
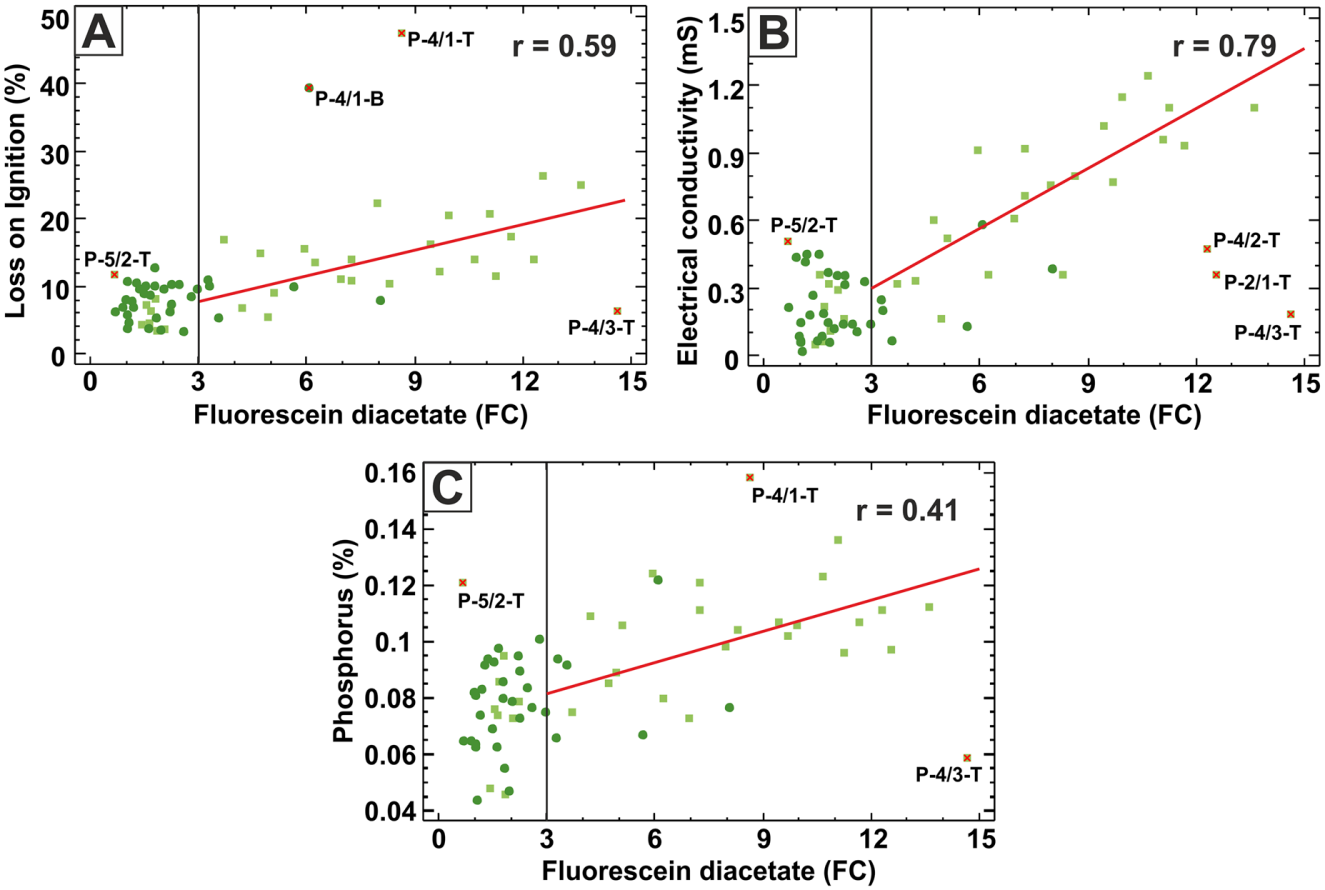
Linear relationship between microbial activity (FDA in units of fluorescein concentration; FC) and **A** organic matter content (loss on ignition), **B** electrical conductivity (EC) and **C** phosphorus concentration. The Pearson’s linear correlation coefficient (*r*) is shown in each plot. Vertical black line at the FDA = 3 FC value separates the two apparent bivariate sample groups. Dark green dots: subsoil samples; light green squares: topsoil samples; red crosses: bivariate outliers marked with their sample identification codes. Note that the topsoil and subsoil samples at the P-4/1 sampling site come from an oxbow lake located in the river terrace

The bivariate analysis, using the least squares linear regression method, is applied to investigate the relationship between the measured soil parameters, contaminants and FDA bulk microbial activity. The FDA vs LOI regression biplot shows the expected overall relationship: increasing organic matter content stimulates higher microbial activity (Fig. 7). However, the more detailed analysis reveals the heterogeneity of processes in the studied floodplain soils. According to Fig. 7, there is no correlation between FDA and LOI for samples having FDA < 3 FC values, while there is a statistically significant linear relationship (*r* = 0.59; 95% confidence level) only in the topsoil samples having FDA > 3 FC values. Electrical conductivity, representing soluble soil salt content, has exactly the same bivariate relationship with FDA (*r* = 0.79 for topsoil samples having FDA > 3 FC value). The strong positive correlation with EC is important because EC defines the activity thresholds for plants and microbes (Doran & Safley, 1997). Just like LOI and EC, phosphorus concentration is related to FDA microbial activity (*r* = 0.41) only for the topsoil samples with FDA value above 3 FC. Typically, the swamp site of an oxbow lake (sample P-4/1) is present as bivariate outlier, and it is not associated with the above identified two bivariate groups.

According to the regression analysis results, FDA microbial activity does not seem to be related to soil pH, CaCO_3_ or any particular grain size.

**Fig. 8:**
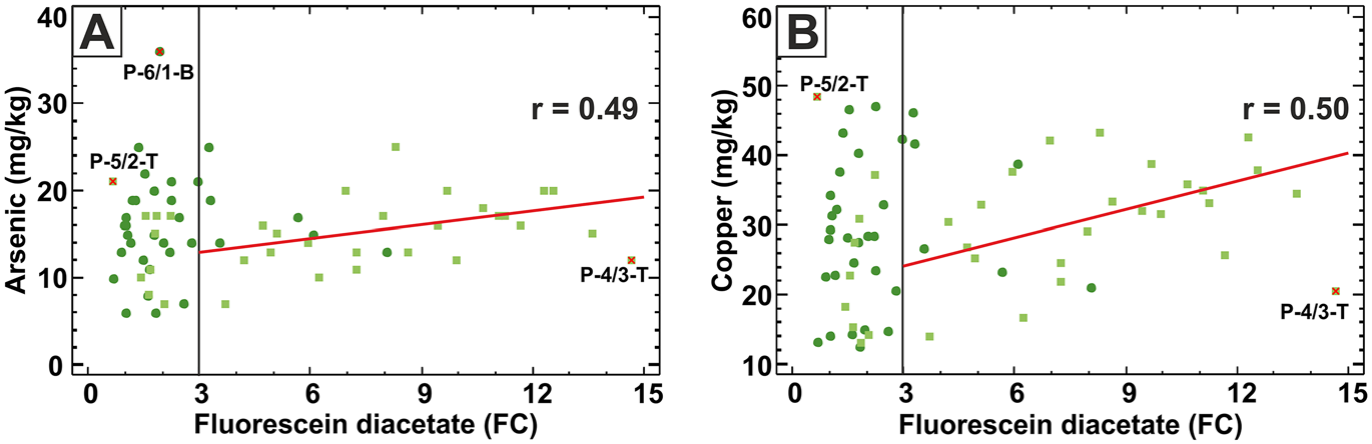
Linear relationship between microbial activity (FDA in units of fluorescein concentration; FC) and PTEs. **A** Arsenic concentration and **B** copper concentration. The Pearson’s linear correlation coefficient (*r*) is shown in each plot. Vertical black line at the FDA = 3 FC value separates the two apparent bivariate sample groups. Dark green dots: subsoil samples; light green squares: topsoil samples; red crosses: bivariate outliers marked with their sample identification codes

The relationship between arsenic and FDA, together with copper and FDA, is very similar to LOI, EC and P: there is no correlation for samples having FDA < 3 FC values, while there is a statistically significant linear relationship (*r* = 0.49 and *r* = 0.50, respectively; 95% confidence level) only in the topsoil samples having FDA > 3 FC values (Fig. 8).

**Fig. 9:**
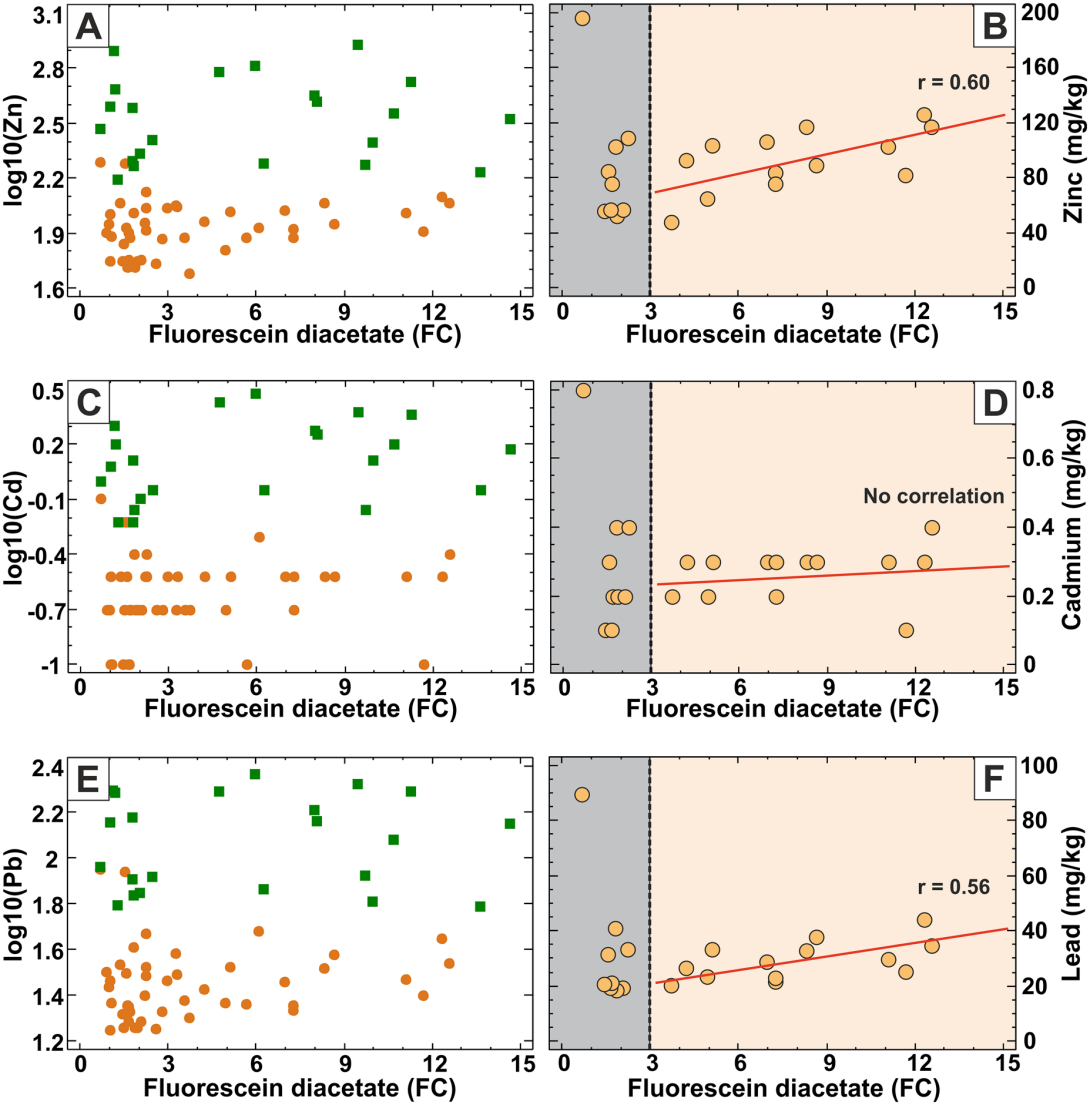
Linear relationship between microbial activity (FDA in units of fluorescein concentration; FC) and PTEs. **A**, **B** Correlation between FDA and zinc concentrations. **C**, **D** Correlation between FDA and cadmium concentrations. **E**, **F** Correlation between FDA and lead concentrations. Note the separation of sample points according to the FDA = 3 FC threshold value (vertical grey dashed line in **B**, **D**, **F**). The Pearson’s linear correlation coefficient (*r*) is shown for samples with FDA > 3 FC value (**B**, **D**, **F**). Green squares: alluvial plain samples; brown dots: river terrace samples

Unlike As and Cu, zinc has no correlation with FDA in the alluvial plain areas at all. However, zinc has a significant relationship (*r* = 0.6) with the bulk microbial activity in the river terrace area, limited to the topsoil zone only, and again where FDA > 3 (Fig. 9A, B). Lead displays exactly the same relationship with FDA (*r* = 0.56) (Fig. 9E, F). The spatially varying relationships among the measured parameters highlight the heterogeneity of the geochemical–microbiological processes in the studied floodplain soils. Despite the clear separation of the alluvial plain and river terrace samples in the Cd-FDA biplot, no correlation is apparent due to the measured very low Cd concentrations (Fig. 9C, D).

In summary, bivariate analysis using the least squares linear regression method revealed that FDA bulk microbial activity is strongly correlated (*r* = 0.41–0.79) with LOI, EC and P and furthermore with As and Cu concentration but only in the topsoil samples where FDA > 3. Correlation with toxic elements implies the apparent contradictory relationship that the increase of these toxic elements promotes the bulk microbial activity in the studied floodplain topsoils. Again, Zn, Cd and Pb have a different behaviour: these PTEs have significant (95% confidence level) linear relationship (*r* = 0.56–0.60) with FDA only in the river terrace topsoils where FDA > 3.

**Fig. 10:**
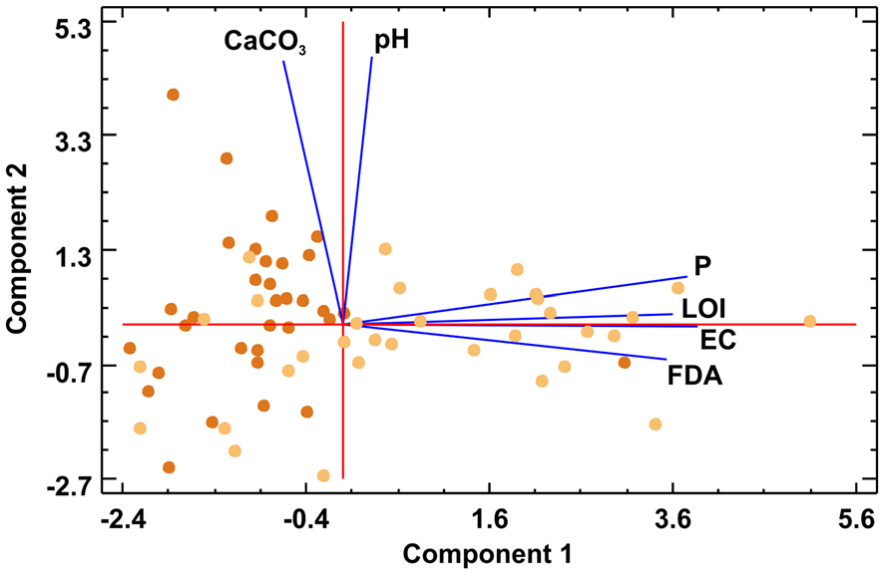
Principal component analysis biplot of the studied parameters, showing their influences on the defined first two components. Dark brown dots: subsoils samples; light brown squares: topsoil samples

PCA of the studied soil parameters revealed two processes (principal components covering 71% of the total variance) related to FDA bulk microbial activity (Table 3). The first principal component (48% of total variance) contains LOI (approximating organic matter content), EC (representing soluble salt content) and the phosphorus concentration, together with FDA, all having the same loadings (around 0.5; Table 4) on this component (Table 4) (Fig. 10). This component can be interpreted as the soil forming process prevailing in the topsoils, which also influences the bulk microbial activity. Note that topsoil and subsoil samples are clearly separated along the first principal component having positive and negative weights in this PCA axis (Fig. 10).

**Table 3:** Principal components with their eigenvalues, percentages of variance and their cumulative percentages. Note that the first two components explain the 71% of total variance (red highlight)

**Component number**	**Eigenvalue**	**Percent of variance**	**Cumulative percentage**
**1**	2.8951	48.252	48.252
**2**	1.3578	22.629	70.881
**3**	0.6887	11.478	82.359
**4**	0.5179	8.632	90.992
**5**	0.35	5.833	96.824
**6**	0.1905	3.176	100

**Table 4:** The influence (loading) of the different parameters on the first two principal components

**Parameter**	**Component 1**	**Component 2**
FDA	0.4768	− 0.0915
LOI	0.4845	0.0266
EC	0.521	− 0.003
pH (water)	0.0431	0.7047
CaCO3	− 0.0881	0.692
P	0.5069	0.1242

The second principal component covering the 23% of the total variance (Table 3) contains the carbonate content and the pH variables, having almost equal loadings (ca. 0.7; Table 4), which represents the carbonate equilibrium process in the studied soils. Subsoil samples tend to have high weights on this PCA axis showing that the pH-sensitive carbonate dissolution is dominant at deeper horizons, however, with no obvious influence on the bulk microbial activity (Fig. 10).

## Discussion

PTE concentrations are elevated above the EQS levels in the whole Drava River floodplain, as also confirmed by this study. The most immediate finding of the present investigation is the horizontal and vertical spatial heterogeneity of the studied floodplain soils and sediments, reflecting well-defined geochemical regimes. These are, horizontally, the regularly flooded active floodplain areas covered by riparian forest and the river terrace (former active floodplain) covered by agricultural lands, while vertically these are the fertile topsoil and the sandy-silty subsoil horizons. The studied soil parameters, PTEs and FDA values systematically correspond to these geochemical regimes but in various ways.

First, soil carbonate content, pH, As and Cu are uniformly distributed all around the studied underground space irrespective of the geomorphological setting (alluvial plain vs river terrace) and soil horizon (topsoil vs subsoil) (see Figs. 2, 3, and 4). This suggests that these chemical parameters are related to the broad and uniform natural geochemical background. Second, not surprisingly, the bulk soil microbial activity as expressed by the FDA activity parameter follows the spatial distribution pattern of organic matter (LOI), dissolved salt (EC) and nutrient (P) which are concentrating in the near-surface topsoil horizon, especially in the active alluvial plain (compare Figs. 2 to 6). The third group of parameters is composed of Zn, Cd and Pb, which have low concentrations (below EQS) at each soil depth level in the river terrace area, while they have very high concentrations in the alluvial plain, especially in topsoils (see Fig. 5). This shows that these contaminants are still impacting the active flood zone above toxic levels, originating from upstream sources such as metal mining and metal industry areas (Halamić et al., 2003; Peh et al., 2008; Šajn et al., 2011).

More exciting is the spatially heterogeneous processes acting between toxic elements and the total microbial activity. First, it seems that the FDA parameter has no obvious correlation with any of the other soil and PTE parameters below the FDA value of 3 FC. Almost all of the samples with FDA < 3 come from the subsoil horizon. The univariate analysis and the box plots clearly show that the total microbial activity, measured by fluorescein diacetate assays, is significantly higher near the surface in the topsoil region. Recent studies also confirmed this heterogeneity regarding the microbial biomass and diversity (Mukhtar et al., 2021; Samec et al., 2020). It was stated that the microbial abundance and the structure of the community are decreasing with depth in a vertical soil profile. However, in deeper horizons, below 2 m, the diversity can be as high or even higher than that in the above laying regions (Kramer et al., 2013; Seuradge et al., 2017; Steger et al., 2019; Van Leeuwen et al., 2017). According to Van Leeuwen et al. (2017), the land use can strongly affect the type and the quantity of microbes in the topsoil layer, with 4–5 times lower biomass in the arable fields than in forests and grasslands. However, the effect of the land use tends to disappear in the deeper soil horizons, below 30 cm. This is confirmed by the present study because FDA have similarly low values in the subsoil horizon irrespective to the geomorphological setting (alluvial plain vs river terrace). Still, the present study cannot find a plausible explanation for the sharp FDA = 3 FC limiting value.

Second, for FDA > 3 FC values (including almost all of the topsoil samples), significant linear correlation (*r* = 0.41–0.79) exists between FDA activity and LOI, EC and P, but CaCO_3_ and pH lack any link to FDA. This is confirmed by the multivariate PCA analysis, whereas the first principal component represents the ‘soil fertility processes’ grouping soil LOI, EC and P, together with FDA activity, and guiding the variability of topsoil samples along this axis (Fig. 10). The second principal component groups carbonate content and pH variables, representing natural soil carbonate equilibrium with no effect on FDA, and guides the variability of subsoil samples along this axis (Fig. 10).

The third main outcome of the investigation is the somewhat unexpected positive relationship between the toxic elements (with As and Cu in the first place) and FDA soil microbial activity (for topsoils where FDA > 3 FC) which is in contradiction to the similar study of Liu et al. (2018). This apparent contradiction is resolved by assuming that some microbe species can adapt to the elevated toxic element concentrations in the studied floodplain topsoils. The few PTE tolerating microbes may take over the other species and proliferate as dominant species in the fertile topsoil horizon.

Finally, it seems that the effect of PTEs on the microbes is specific and strongly depends on geochemical conditions. The FDA activity is positively correlated with the PTEs in the river terrace topsoils (Cd is an exception due to its low concentrations); however, only As and Cu exert influence on the soil microbial community in the active alluvial plain topsoils. This suggests that in this geochemical regime, As and Cu are present in soluble forms, bioavailable for microbes, while Zn and Pb (and Cd) are not available, most probably due to their binding to organic matter or being bound as sulphides in the frequently flooded, organic-rich reducing alluvial plain topsoils.

## Conclusions

The historical mining and smelting, just like other industrial activities, left their footprints on the Drava River floodplain. Elevated toxic element concentrations can be measured throughout the whole floodplain in subsoils (sediments) and topsoils, exceeding the EQS thresholds. Fluorescein diacetate (FDA) test seems to be a useful tool for studying the local microbial activities; however, it has to be coupled with other analyses such as soil respiration test (Hashimoto et al., 2015) or multi-SIR profiles of soil microbial communities test (Mucsi et al., 2021) to reach sufficient results for investigating toxic effect of PTEs in floodplain sediments and soils. The FDA test was developed as a fast and inexpensive indicator for assessing the fertility of agricultural soils; hence, the best results can be retrieved for local processes. With respect to the relationship between PTE contamination and bulk microbial activity measured by FDA, it can be concluded that this method is applicable only within limitations. The results were relevant for essential nutrients (e.g. phosphorus), but for a heavy metal stress the microbial activity has a different reaction. The statistically significant positive correlations in bivariate regression analysis let us conclude that the microbes can adapt to the changes in toxic element concentrations, or at least a few species can highly tolerate it, so the overall cell number does not decrease, only the diversity or the proportion of some species. The microbial activity results did not reflect the obvious geochemical difference between the alluvial plain and river terrace areas. Thus, fluorescein diacetate test alone may not be suitable for investigating and monitoring the toxic effect of recent and historical contamination in floodplain sediments because of the adaptation of some microbial physiological groups.

## Data Availability

Data sharing is not applicable to this article as no datasets were generated or analysed during the current study.
